# Genome-Wide Identification and Expression Pattern Analysis of the Phenylalanine Ammonia-Lyase (PAL) Gene Family in Wheat Under Stripe Rust

**DOI:** 10.3390/biology15191745

**Published:** 2026-10-02

**Authors:** Niannian Yan, Shuangqing He, Dongqing Ding, Qingyu Zhang, Qi Liu

**Affiliations:** 1 Key Laboratory of the Pest Monitoring and Safety Control of Crops and Forests of the Xinjiang Uygur Autonomous Region, College of Agronomy, Xinjiang Agricultural University, Urumqi 830052, China; 17690712604@163.com (N.Y.); h200208152022@163.com (S.H.); 13201239058@163.com (D.D.); 18837863651@163.com (Q.Z.); 2 Key Laboratory of Prevention and Control of Invasive Alien Species in Agriculture & Forestry of the North-Western Desert Oasis, Ministry of Agriculture and Rural Affairs, Urumqi 830052, China

**Keywords:** wheat, wheat stripe rust, phenylalanine ammonia-lyase, gene family, expression pattern

## Abstract

Phenylalanine ammonia-lyase (PAL) has been identified as the key rate-limiting enzyme of the phenylpropanoid metabolic pathway, which affects the synthesis of plant secondary metabolites and stress responses. To investigate the composition and function of the PAL gene family in wheat against wheat stripe rust, 54 *TaPAL* gene family members were identified based on the wheat IWGSC RefSeq v2.1 genome data. Phylogenetic analysis of PAL proteins from wheat, maize, rice, tobacco, and Arabidopsis indicated that wheat PAL genes are more closely related to those of maize and rice. Promoter analysis revealed that these genes contain multiple hormone- and stress-responsive cis-acting elements. To investigate the expression characteristics of the *TaPAL* family under diverse stress conditions, qRT-PCR was conducted to analyze the transcriptional responses of 24 members distributed across different chromosomes to *Puccinia striiformis* f. sp. *tritici* (*Pst*) infection. Under *Pst* infection, relatively low basal expression was maintained by most *TaPAL* genes during early stages, whereas most *TaPAL* genes were significantly induced at 120 hpi. High expression levels were sustained by most genes at 240 hpi, and decreases were observed at 360 hpi. Four genes selected based on our laboratory’s previous transcriptome data were used for expression analysis under exogenous hormone treatments and abiotic stresses. Diverse expression patterns were exhibited by the *TaPAL* genes under hormone treatments and abiotic stresses, indicating possible regulatory differentiation among family members. The results provide candidate genes for further functional studies of the wheat PAL gene family in stress responses.

## Simple Summary

In this study, 54 *TaPAL* genes were identified from the wheat genome. Bioinformatic analysis revealed that the encoded proteins possessed similar gene structures, conserved domains, and cytoplasmic localization. Phylogenetic analysis indicated that they were closely related to the PAL genes of maize and rice and were widely distributed throughout the genome. The promoter regions of *TaPAL* genes were found to be enriched with multiple responsive elements. These genes were significantly upregulated at 120 hpi following *Puccinia striiformis* f. sp. *tritici* infection and were differentially induced by various hormones and abiotic stresses. The results provide candidate genes for further functional studies of the wheat PAL gene family in stress responses.

## 1. Introduction

Wheat (*Triticum aestivum*) is one of the most important cereal crops globally [1,2]; however, its production is frequently threatened by various pathogens. Wheat stripe rust is attributed to *Puccinia striiformis* f.sp. *tritici*, designated as a wind-borne fungal disease [3], and established as one of the principal restricting factors undermining global wheat production stability and productivity in the 21st century [4,5].

Currently, disease resistance breeding is primarily dependent on resistance genes; however, due to the frequent variation in *Pst* physiological races, variety resistance is easily lost, and available resistance gene resources are extremely limited [6]. Therefore, the mining of key disease resistance genes and the systematic characterization of their physicochemical properties and phylogenetic relationships were carried out, contributing to the in-depth dissection of the wheat–Pst interaction mechanism, and a significant theoretical basis was established for the breeding of persistent disease-resistant cultivars and the formulation of green management strategies.

Plant metabolic processes were generally classified as either primary or secondary, and the phenylpropanoid pathway ranked among the most important pathways in secondary metabolism [7]. The phenylpropanoid metabolic pathway enabled plants to cope with biotic stress by synthesizing secondary metabolites including lignin, flavonoids, and phytoalexins. Such metabolites enhance cell wall integrity, directly inhibiting pathogen propagation, and facilitate the transduction of defense signals [8]. Phenylalanine ammonia-lyase (PAL, EC 4.3.1.5) was identified as the enzyme that catalyzed the first step of the phenylpropanoid pathway, in which phenylalanine was non-oxidatively deaminated to form trans-cinnamic acid [9], subsequently regulating the synthesis of downstream lignin, flavonoids, and disease resistance-related substances [10,11]. PAL was found to be widely distributed in various plants and was identified in Arabidopsis (*Arabidopsis thaliana*) [12], tobacco (*Nicotiana tabacum* L.) [13], maize (*Zea mays* L.) [14], rice (*Oryza sativa* L.) [15], sorghum (*Sorghum bicolor* L.) [16], and wheat (*Triticum aestivum* L.) [17]. The PAL gene family was found to contain multiple members, each of which was shown to exhibit a unique expression pattern and to generate differential responses to biotic and abiotic stresses [18]. In wheat, the full complement of 54 *TaPAL* members was identified at the genomic level, and the expression patterns of this family under stripe rust and salt stress were preliminarily investigated in previous studies [17,19]; however, under stripe rust stress, the expression of only five *TaPAL* genes was analyzed at the early infection stages (6–48 h after inoculation), and the expression responses and long-term dynamics of the *TaPAL* family at the late stages of *Pst* infection remain unclear.

High-quality reference genome sequences and their annotations for multiple plant species, such as wheat, were successively released and were progressively refined through continuous updates, thereby providing resources for the systematic study of gene families such as the PAL family [20]. In this study, an expanded qRT-PCR analysis was conducted to profile the transcriptional responses of 24 *TaPAL* members distributed across different chromosomes to *Pst* infection at seven time points from 0 to 360 hpi, as well as to exogenous hormone treatments and abiotic stresses. The expression dynamics of these genes under diverse stress conditions were systematically compared, and their possible regulatory patterns in wheat stress responses were explored, with the aim of providing candidate genes for further functional studies of the *TaPAL* gene family.

## 2. Materials and Methods

### 2.1. Identification of PAL Gene Members in Wheat

To identify the members of the wheat PAL gene family, the DNA FASTA and GFF3 files of the wheat IWGSC RefSeq v2.1 genome were first downloaded from URGI (https://wheat-urgi.versailles.inra.fr/Seq-Repository/Assemblies, accessed on 28 May 2025) DNA FASTA sequence files and GFF3 genome annotation files for rice (*Oryza sativa* L.), maize (*Zea mays* L.), *Arabidopsis* (*Arabidopsis thaliana*), and tobacco (*Nicotiana tabacum* L.) were downloaded from Ensemble Plants (https://plants.ensembl.org/index.html, accessed on 28 May 2025). Using the reported PAL protein sequences as queries, the initial BLAST (e-value ≤ 1 × 10^−10^) analysis was performed against the IWGSC RefSeq v2.1 genome using TBtools version 2.435 software (https://github.com/CJ-Chen/TBtools, accessed on 28 May 2025) [21]. The Hidden Markov Model (HMM) profile of the PAL domain (PF00221) was downloaded from the InterPro database (https://www.ebi.ac.uk/interpro/, accessed on 30 May 2025) [22]. PAL family members were identified by searching the wheat genome sequences using the HMM profile via the Simple HMM Search tool in TBtools version 2.435 software (e-value ≤ 1 × 10^−10^). After two rounds of search results were merged and redundant sequences were removed, the sequences of *TaPAL* candidate genes in wheat were analyzed using the NCBI-CDD (https://www.ncbi.nlm.nih.gov/Structure/bwrpsb/bwrpsb.cgi, accessed on 30 May 2025) database to further verify whether they contained conserved domains or not [23].

### 2.2. Phylogenetic Analysis of the PAL Gene Family

A total of 4 AtPAL, 9 OsPAL, 18 ZmPAL, 2 OisPAL and 54 TaPAL proteins were selected for phylogenetic analysis (in Table S1). The protein sequences of wheat, Arabidopsis, rice, maize, and tobacco PAL genes were compared by multiple sequence alignment using the software MEGA 11.0. The phylogenetic tree was constructed using the Maximum Likelihood (ML) method (1000 bootstrap trials). The phylogenetic tree was visualized with iTOL (IToL, version 7, https://itol.embl.de/, accessed on 5 June 2025).

### 2.3. Gene Structure, Conserved Domain, and Conserved Motif Analysis of Wheat TaPAL

The coding sequence of 54 *TaPAL* gene was aligned with its genomic sequence using TBtools (version 2.435) to construct a gene structure map. The motifs of all PAL protein sequences were identified using the online software MEME 5.5.9 (Multiple EM for Motif Elicitation, https://meme-suite.org/meme/, accessed on 5 June 2025). Conserved domains within the PAL family were identified using the Pfam database, with confirmation from the NCBI Conserved Domain Database (https://www.ncbi.nlm.nih.gov/Structure/bwrpsb/bwrpsb.cgi, accessed on 5 June 2025).

### 2.4. Analysis of Cis-Elements in the Promoter of TaPAL Genes

The 2 kb promoter region sequences upstream of the *TaPAL* gene were extracted using TBtools version 2.435 software and submitted to the PlantCARE database (http://bioinformatics.psb.ugent.be/webtools/plantcare/html, accessed on 8 June 2025) [24] for cis-element prediction and the results were visualized using TBtools version 2.435 software.

### 2.5. Chromosomal Localization and Collinearity Analysis

All identified *TaPAL* genes were analyzed by aligning their sequences with the corresponding genome reference GFF3 file (IWGSC V 2.1) to extract the start- and end-location information of the genes. The chromosomal locations were determined by visualizing the results using TBtools version 2.435 software (https://github.com/CJ-Chen/TBtools-II/releases, accessed on 12 June 2025). Interspecies and intraspecies collinearity were analyzed using the Multicollinearity Scanning Toolkit X [25] and visualized with TBtools version 2.435 software.

### 2.6. Plant Materials and Treatments

Wheat cultivars Xinchun 32 (XC 32) and Minxian 169 (MX 169), as well as *Puccinia striiformis* f. sp. *tritici* (*Pst*), were provided by the Laboratory of Plant Disease Epidemiology, College of Agronomy, Xinjiang Agricultural University. XC 32 was developed in Xinjiang and exhibits high resistance to the currently epidemic *Pst* races (CYR33, CYR34) [26]. The *Pst* was expanded and preserved by MX 169. To investigate the tissue-specific expression level of the *TaPAL* gene, leaves, stems, and roots were collected separately from wheat seedlings at the two-leaf stage. To analyze the biotic and abiotic stress responses, mixed *Pst* strains were artificially inoculated onto XC 32 seedlings at the two-leaf stage of wheat. The inoculated wheat plants were placed in a growth chamber and incubated at 80% humidity and 11–13 °C in the dark for 24 h, and subsequently transferred to an environment of 11–13 °C with a photoperiod of 16 h light/8 h dark. Wheat leaf samples were collected at 0, 12, 24, 48, 120, 240 and 360 h post-inoculation (hpi). Simultaneously, XC 32 seedlings at the two-leaf stage of wheat were treated with 100 mmol/L ethephon (ETH), 100 mmol/L abscisic acid (ABA) and 2 mmol/L salicylic acid (SA), respectively. Drought stress was induced by 20% (*w*/*v*) PEG 6000. Salt stress was imposed by 200 mmol/L NaCl solution. Wounding treatment was performed by cutting leaves with a blade. Cold stress was applied by placing seedlings in a growth chamber at 4 °C. Wheat samples were collected at 12, 24, 48, 72, 96 and 120 h after each treatment. For *Pst* inoculation, the samples collected at 0 hpi were used as the experimental control; for hormone treatments and abiotic stresses, the untreated samples collected at 0 h (before treatment) were used as the experimental control; three independent biological replicates were established for each treatment and time point. All leaf samples were immediately frozen in liquid nitrogen and stored at −80 °C for subsequent use.

### 2.7. qRT-PCR Analysis

Total RNA was extracted using the Plant Total RNA Isolation Kit Plus (FOREGENE, RE-05021, Chengdu, China). First-strand cDNA synthesis was carried out using the All-in-One First-Strand Synthesis MasterMix (Yugong Biotech, Lianyungang, China). All primers were designed based on gene sequences identified by SnapGene version 6.0.2 software and synthesized by Xinjiang Youkang Biotech (Urumqi, China), and their sequences are provided in Table S4. The qRT-PCR was conducted using Taq SYBR^®^ Green qPCR Premix (Yugong Biotech, Lianyungang, China) on a CFX96 Real-Time System (Bio-Rad, Hercules, CA, USA). Each 20 μL reaction mixture contained 10 μL of Taq SYBR^®^ Green qPCR Premix, 0.4 μL of each primer, 1 μL of the cDNA template, and 8.2 μL of nuclease-free water. The thermal cycling program consisted of an initial denaturation at 95 °C for 30 s, followed by 40 cycles of denaturation at 95 °C for 10 s, annealing at 60 °C for 10 s, and extension at 72 °C for 30 s. All genes were detected using three biological replicates and three technical replicates. The *Ta26S* [27,28] gene was used as an internal reference to normalize the relative expression of the candidate genes, and the mRNA expression levels were calculated using the 2^^−ΔΔCT^ method [29]. Statistical significance of differences among treatments was determined by two-way ANOVA followed by Dunnett’s and Tukey’s multiple comparisons test (*p* < 0.05) in GraphPad Prism version 10.1.2.

## 3. Result

### 3.1. Identification of TaPAL Genes in Wheat

In this study, 54 members of the *TaPAL* family genes were eventually identified and named *TaPAL1* to *TaPAL54* (in Table S2). The family members were confirmed to possess the conserved PAL domain based on the results obtained from the NCBI-CDD (https://www.ncbi.nlm.nih.gov/Structure/bwrpsb/bwrpsb.cgi, accessed on 5 June 2025) and Inter Pro (http://www.ebi.ac.uk/interpro/scan.html, accessed on 5 June 2025). The details of the genes/proteins are listed in Table S3. The amino acid lengths of the *TaPAL* members ranged from 132 to 714 amino acids (aa); the theoretical isoelectric point (PI) values ranged from 5.43 to 7.19; and the molecular weights ranged from 14.67 kDa to 77.31 kDa. The predicted subcellular localization showed that 54 TaPAL proteins were localized in the cytoplasm. As shown in Figure 1a, a total of 54 *TaPAL* genes were identified in this study, and two domains were contained, namely the PLN02457 and Lyase_I_like superfamily. The results of the gene structure analysis are shown in Figure 1b, in which 17 *TaPAL* genes were found to contain only CDS regions, while 37 *TaPAL* genes were identified as containing both CDS and UTR regions. The conserved motif results of TaPAL proteins are shown in Figure 1c, and a total of 10 motifs were identified in the *TaPAL* gene family. These results indicate that most *TaPAL* gene family members were found to possess identical gene structures and conserved domains, suggesting that they are highly similar in both function and sequence.

### 3.2. Phylogenetic Analysis of TaPAL Genes

The results of the phylogenetic analysis of the PAL family are shown in Figure 2. A total of 87 PAL members were identified in five species: 54 in wheat, 18 in maize, 9 in rice, 2 in tobacco, and 4 in Arabidopsis. All full-length amino acid sequences of the PAL members were used to construct a phylogenetic tree to determine the evolutionary relationships of the *TaPAL* genes in wheat, maize, rice, tobacco, and Arabidopsis and to further classify the PAL members. Consequently, the PAL members were divided into four groups based on the phylogenetic analysis. The *TaPAL* genes had corresponding sequences in each group, where maize was mainly classified into groups II, III, and IV; rice was mainly classified into groups I, II, and III; and tobacco and Arabidopsis were mainly classified as group II. These results indicate that the *TaPAL* genes in wheat were more closely related to those in maize and rice than to those in tobacco and Arabidopsis.

### 3.3. Analysis of Cis-Regulatory Elements in the Promoter Region of TaPAL Genes

Cis-acting element prediction was performed on the 2000 bp upstream promoter sequences of 54 wheat *TaPAL* genes, and the results are shown in Figure 3. A total of 36 types of cis-regulatory elements were identified, which were classified into four functional categories: light-responsive, hormone-regulated, abiotic/biotic stress-responsive, and tissue- and development-specific regulatory elements. Among the promoter sequences of all *TaPAL* family members, the four most abundant elements were various light-responsive cis-elements, the abscisic acid-responsive element ABRE, and the methyl jasmonate-responsive elements TGACG-motif and CGTCA-motif. The results indicate that light signals and multiple hormone signals could be integrated by the wheat *TaPAL* family to jointly mediate the responses of wheat to environmental stresses and regulate its growth and development processes.

### 3.4. Chromosomal Distribution of TaPAL Genes

Based on the gene annotation information, 54 *TaPAL* genes were located on the IWGSC Refseq V2.1 chromosome. The 54 genes were found to be widely distributed across the whole genome. As shown in Figure 4, chromosomes 2A and 2B were found to contain the largest number of *TaPAL* genes, with eight genes each; this was followed by chromosome 2D, on which seven genes were distributed. Six and four genes were detected on chromosomes 1B and 1D, respectively; three genes were identified on each of the chromosomes 1A, 6A, 6B and 6D; two genes were observed on each of the chromosomes 3B, 3D and 5B; while only one gene was distributed on each of the chromosomes 3A, 4A and 5A.

### 3.5. Collinearity Analysis of TaPAL Gene Family

To explore the evolutionary process of the *TaPAL* gene family, intraspecies collinearity (shown in Figure 5a) and interspecies collinearity (shown in Figure 5b) were analyzed. Intraspecies collinearity analysis detected eight pairs of collinear genes among the 54 *TaPAL* genes. The results showed that two pairs were located on chromosomes 1A/1B, three pairs were located on chromosomes 1A/1D, and three pairs were located on chromosomes 1B/1D, indicating that the allelic genes on Chr1 were highly conserved during evolution, and these gene pairs were inferred to be subgenome homoeologous genes.

Interspecies collinearity further revealed the evolutionary relationships between the PAL gene family and other species. As shown in Figure 5b, eleven pairs of collinear genes were identified between *Triticum aestivum* L. and *Zea mays* L., and seven pairs of collinear genes were found between *Triticum aestivum* L. and *Oryza sativa* L., whereas only one pair of collinear genes was detected between *Triticum aestivum* L. and *Nicotiana tabacum* L., and no collinear genes were found between *Triticum aestivum* L. and *Arabidopsis thaliana*. Based on this analysis, the PAL members between *Triticum aestivum* L. and *Zea mays* L. or *Oryza sativa* L. showed high consistency and were more closely related, compared to those in *Arabidopsis thaliana* and *Nicotiana tabacum*.

### 3.6. Expression Pattern Analysis of TaPAL Genes in Wheat–Pst Interactions

To comprehensively evaluate the transcriptional responses of the *TaPAL* gene family to stripe rust infection, 24 members distributed across different chromosomes were selected, and qRT-PCR analysis was conducted at seven time points (0, 12, 24, 48, 120, 240 and 360 hpi). As shown in Figure 6, relatively low transcript levels were maintained by most *TaPAL* genes during the early infection stages (0–48 hpi). However, during the later stages of infection, obvious induced expression was observed for most *TaPAL* genes at 120 hpi. Distinct temporal dynamic characteristics were exhibited by these PAL members distributed across different chromosomes. Expression peaks at 120 hpi were reached by a subset of genes (including *TaPAL9*, *TaPAL28*, *TaPAL42*, *TaPAL47*, *TaPAL38*, *TaPAL22*, *TaPAL33*, *TaPAL23*, *TaPAL44* and *TaPAL39*), followed by declines at 240 hpi. By contrast, high expression levels at 240 hpi were maintained by most members (such as *TaPAL25*, *TaPAL29*, *TaPAL1*, *TaPAL4*, *TaPAL37*, *TaPAL54*, *TaPAL17* and *TaPAL43*), and peak expression was reached by several members at this time point. The results indicate that predominant activation of the wheat PAL gene family was observed during the late stages of *Pst* infection, and that the presence of both conserved family-wide responsiveness and subgenome-specific functional divergence in the reaction to stripe rust was suggested by distinct temporal expression patterns among chromosomally dispersed members.

### 3.7. Tissue Specificity of TaPAL Gene Expression

To investigate the tissue-specific expression patterns of *TaPAL* genes, four members were selected from the aforementioned 24 *TaPAL* genes based on our laboratory’s previous transcriptome data, and their expression levels in root, stem, and leaf were examined. As shown in Figure 7, distinct tissue-specific expression profiles were exhibited by the four genes. Relatively low expression of *TaPAL28* was detected in the root, whereas slightly higher but statistically non-significant expression levels were observed in the stem and leaf. By contrast, extremely significant upregulation of *TaPAL47* in the stem was observed compared with that in the root and leaf. Stable and relatively low expression levels of *TaPAL33* across all three tissues were detected. Additionally, extremely significant upregulation of *TaPAL34* in the leaf was observed compared with that in the root and stem. The results indicate that distinct tissue-specific expression patterns were possessed by different *TaPAL* members.

### 3.8. Expression Pattern Analysis of TaPAL Genes in Response to Hormone Treatments

To investigate whether the *TaPAL* genes were induced by hormone signaling, wheat seedlings were treated with three phytohormones, namely salicylic acid (SA), ethephon (ETH), and abscisic acid (ABA), and the expression levels of the *TaPAL* genes during the wheat response to hormone signaling were subsequently detected. As shown in Figure 8, *TaPAL47* was extremely significantly upregulated after ABA treatment at 12 h and 96 h, whereas no significant changes were observed in *TaPAL28*, *TaPAL33*, and *TaPAL34* at any time after ABA treatment. *TaPAL47* was significantly upregulated after ETH treatment at 120 h, whereas no significant changes were observed in *TaPAL28*, *TaPAL33*, and *TaPAL34* at any time after ETH treatment. *TaPAL28* was extremely significantly upregulated after SA treatment at 24 h, and its expression level was subsequently decreased to a normal level at any other time. *TaPAL34* was extremely significantly upregulated after SA treatment at 24 h, whereas no significant changes were observed in *TaPAL33* and *TaPAL47* at any time after SA treatment. It was indicated that distinct differences were observed in the responses of different *TaPAL* family members to salicylic acid, ethephon, and abscisic acid.

### 3.9. Expression Pattern Analysis of TaPAL Genes in Response to Abiotic Stress

To investigate the expression patterns of *TaPAL* genes under abiotic stress, wheat seedlings were subjected to cold treatment, wounding treatment, NaCl treatment, and PEG6000 treatment, respectively. As shown in Figure 9, *TaPAL28* was extremely significantly upregulated at 12 h and 24 h after cold treatment, and significantly upregulated at 72 h; *TaPAL47* was significantly upregulated at 12 h after cold treatment, whereas no significant changes were observed in *TaPAL33* and *TaPAL34* at any time after cold treatment. *TaPAL28* and *TaPAL33* were extremely significantly upregulated at 24 h after wounding treatment, whereas no significant changes were observed in *TaPAL34* at any time after wounding treatment. Additionally, *TaPAL47* was significantly upregulated at 24 h after wounding treatment. *TaPAL28* was extremely significantly upregulated at 24 h after NaCl treatment; *TaPAL34* was extremely significantly upregulated at 24 h after NaCl treatment; and *TaPAL47* was significantly upregulated at 12 h after NaCl treatment, and its expression level peaked at 24 h. By contrast, no significant changes were observed in *TaPAL33* at any time after NaCl treatment. *TaPAL28* was significantly upregulated at 24 h after PEG6000 treatment, and its expression level was subsequently decreased to a normal or lower level at the other time points; however, no significant changes were observed in *TaPAL33*, *TaPAL34*, and *TaPAL47* at any time after PEG6000 treatment. It is indicated by these results that significant specificity and differences were exhibited in the responses of different *TaPAL* members to various abiotic stresses, and functional divergence among members of this gene family is accordingly speculated.

## 4. Discussion

The defense of wheat against *Puccinia striiformis* f. sp. *tritici* (*Pst*) is mediated by the synergistic action of multiple genes, a substantial number of which have been characterized as stripe rust resistance genes. Phenylalanine ammonia-lyase (PAL) was identified as the key rate-limiting enzyme of the phenylpropanoid pathway and was shown to contribute substantially to plant biotic stress responses. The complete set of 54 *TaPAL* genes in hexaploid wheat was previously identified by Zhan et al. [17], and fundamental bioinformatic analyses were performed by this group, including phylogeny, chromosomal distribution, gene structure, and cis-element prediction. *TaPAL* expression alterations upon infection with *Fusarium graminearum*, *Puccinia striiformis*, and powdery mildew were uncovered through their transcriptome survey, and it was further demonstrated by VIGS assays that *TaPAL32* and *TaPAL42* positively regulated wheat stripe-rust resistance. Nevertheless, although five *TaPAL* members were validated by qRT-PCR under stripe rust stress and the sampling covered the early infection stage (6–48 hpi) in that study, the long-term expression dynamics of the family at the late infection stages remain to be explored. The transcriptional responses of the *TaPAL* family over the full disease progression, especially at late infection stages, were not experimentally characterized. Second, how *TaPAL* genes responded to phytohormone signals and diverse abiotic stresses was not explored. In the current study, qRT-PCR profiling was performed for 24 *TaPAL* members distributed across different chromosomes, and the sampling time-course was extended to 360 hpi to investigate gene expression throughout the whole *Puccinia striiformis* f. sp. *tritici* infection process. It was revealed that most *TaPAL* genes maintained low basal expression at early infection stages but underwent broad and strong transcriptional induction starting from 120 hpi, indicating that transcriptional activation of this family was triggered during the late phase of stripe rust infection. The inconsistent temporal expression patterns between our data and the earlier report were most likely attributable to differences in wheat cultivar, *Pst* race, and sampling time windows. Beyond biotic stress, expression changes in *TaPAL* genes under exogenous hormone treatments and multiple abiotic stresses were further characterized, and possible regulatory divergence among *TaPAL* members was suggested.

Phenylalanine ammonia-lyase (PAL) was predominantly found in plants and fungi, and was identified as the first rate-limiting enzyme in the phenylpropanoid metabolic pathway [30]. It was shown to regulate the biosynthesis of lignin, flavonoids, isoflavones, alkaloids, and other substances, and therefore, was suggested to play an important role in plant resistance to biotic and abiotic stresses [31]. Regulatory effects on plant growth and development, as well as on resistance to biotic and abiotic stresses, were exerted through the application of exogenous plant hormones, whereby certain developmental processes were promoted or inhibited and plant stress resistance was enhanced [32]. It was reported that after exogenous salicylic acid was applied to grapes, PAL activity, protein synthesis, and gene expression were activated [33]. After maize was treated with ethephon, the activity and transcription level of phenylalanine ammonia-lyase (PAL) were significantly increased, and lignin accumulation was promoted [34]. Differential expression of *Arabidopsis thaliana* PAL genes was observed under drought and salt stress; *AT3G47660* was significantly upregulated under salt stress, whereas *AT5G04230* was significantly down-regulated under drought stress but upregulated under salt stress [35]. It was demonstrated that distinct differences were observed in the responses of different *TaPAL* members to hormones and stress signals. *TaPAL47* was mainly induced by ABA and ETH, whereas *TaPAL28* and *TaPAL34* were primarily responsive to SA. No significant response to any of the three hormones was detected for *TaPAL33*. Under abiotic stress conditions, *TaPAL28* was significantly upregulated by cold, wounding, NaCl, and PEG6000. *TaPAL47* was mainly responsive to cold and NaCl stress; *TaPAL34* was induced by NaCl stress; and *TaPAL33* was only responsive to mechanical wounding. Therefore, divergent expression patterns were exhibited by the four *TaPAL* genes under hormones and stress treatments, and significant functional divergence among members of this gene family was accordingly hypothesized.

In this study, genome-wide identification and systematic bioinformatic analysis of the *TaPAL* gene family were performed based on the IWGSC RefSeq v2.1 genome. Expression profiling of *TaPAL* genes under *Puccinia striiformis* f. sp. *tritici* infection, hormone treatments, and abiotic stresses was conducted by qRT-PCR, and obvious differences among distinct family members in response to biotic and abiotic stresses were revealed. Therefore, significant functional divergence among different *TaPAL* genes was speculated, and distinct roles of individual members in wheat stripe rust resistance and stress adaptation were hypothesized. However, functional validation by virus-induced gene silencing (VIGS) and overexpression techniques was not performed, and the molecular interaction mechanisms between *TaPAL* and *Pst* remained unresolved. In future studies, the stripe rust resistance functions of *TaPAL* genes will be further elucidated, and virus-induced gene silencing (VIGS), overexpression, yeast two-hybrid (Y2H), and co-immunoprecipitation (CoIP) techniques will be employed to dissect the phenylpropanoid pathway-mediated disease resistance regulatory network.

## 5. Conclusions

In this study, a total of 54 *TaPAL* gene family members were identified based on the IWGSC RefSeq v2.1 genome, and their physicochemical properties, gene structures, phylogenetic relationships, chromosomal locations, collinearity, and promoter cis-acting elements were systematically analyzed. The results indicated that *TaPAL* family members were highly conserved in structure and were closely related to PALs from maize and rice, and that a large number of light-responsive, hormone-responsive, and stress-responsive elements were enriched in the promoter regions. Expression pattern analysis showed that the *TaPAL* genes were significantly upregulated at the late stages of infection following *Puccinia striiformis* f. sp. *tritici* inoculation, and that obvious differences were observed in the responses of different members to hormones (SA, ETH, and ABA) and abiotic stresses (cold, wounding, NaCl, and PEG6000). The evolutionary and expression characteristics of the wheat *TaPAL* family were systematically elucidated by this study, and candidate genes were provided for further functional studies of wheat disease resistance.

## Figures and Tables

**Figure 1:**
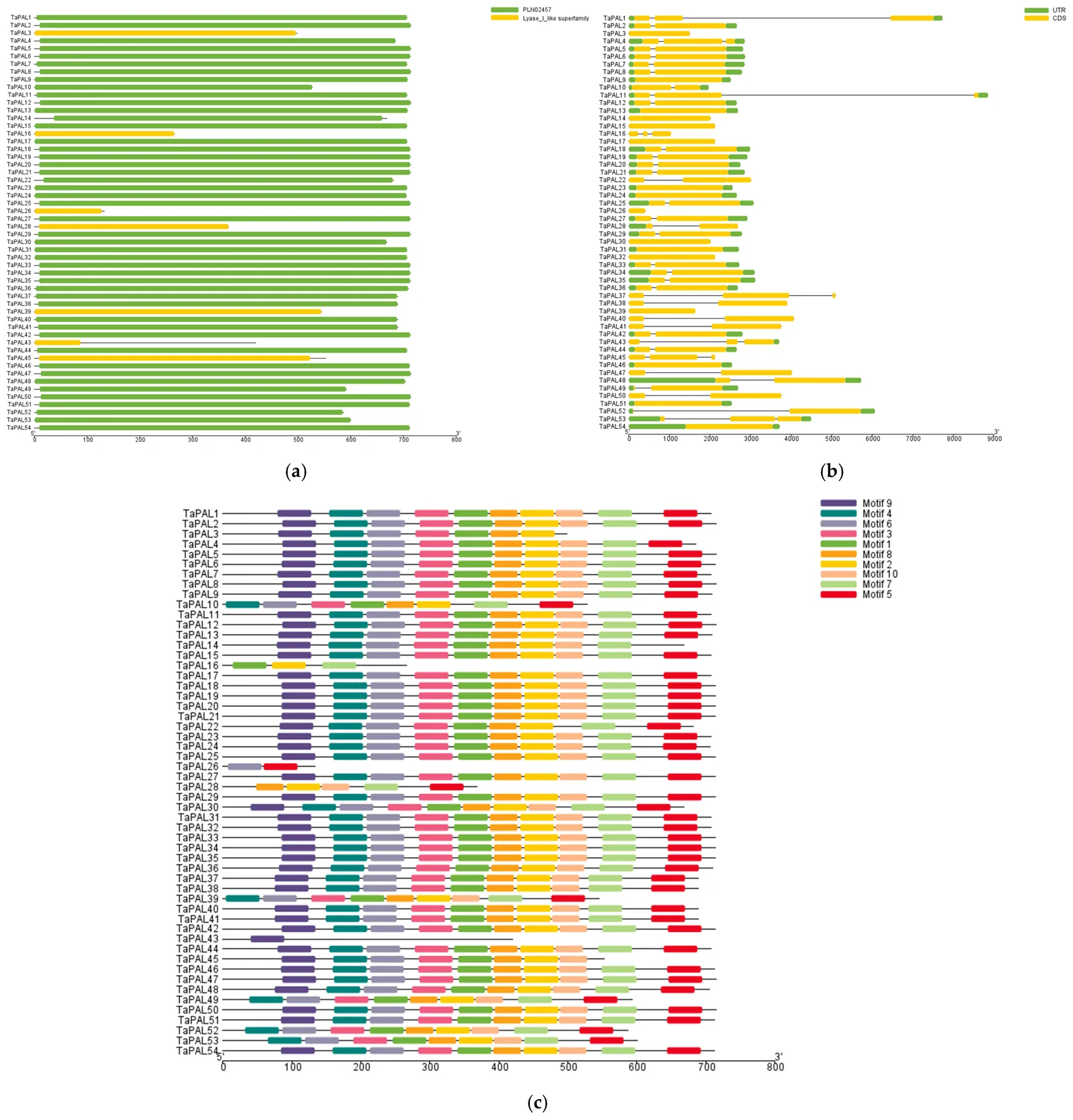
(**a**) Conserved domains; (**b**) gene structure; (**c**) conserved motifs of wheat TaPAL proteins.

**Figure 2:**
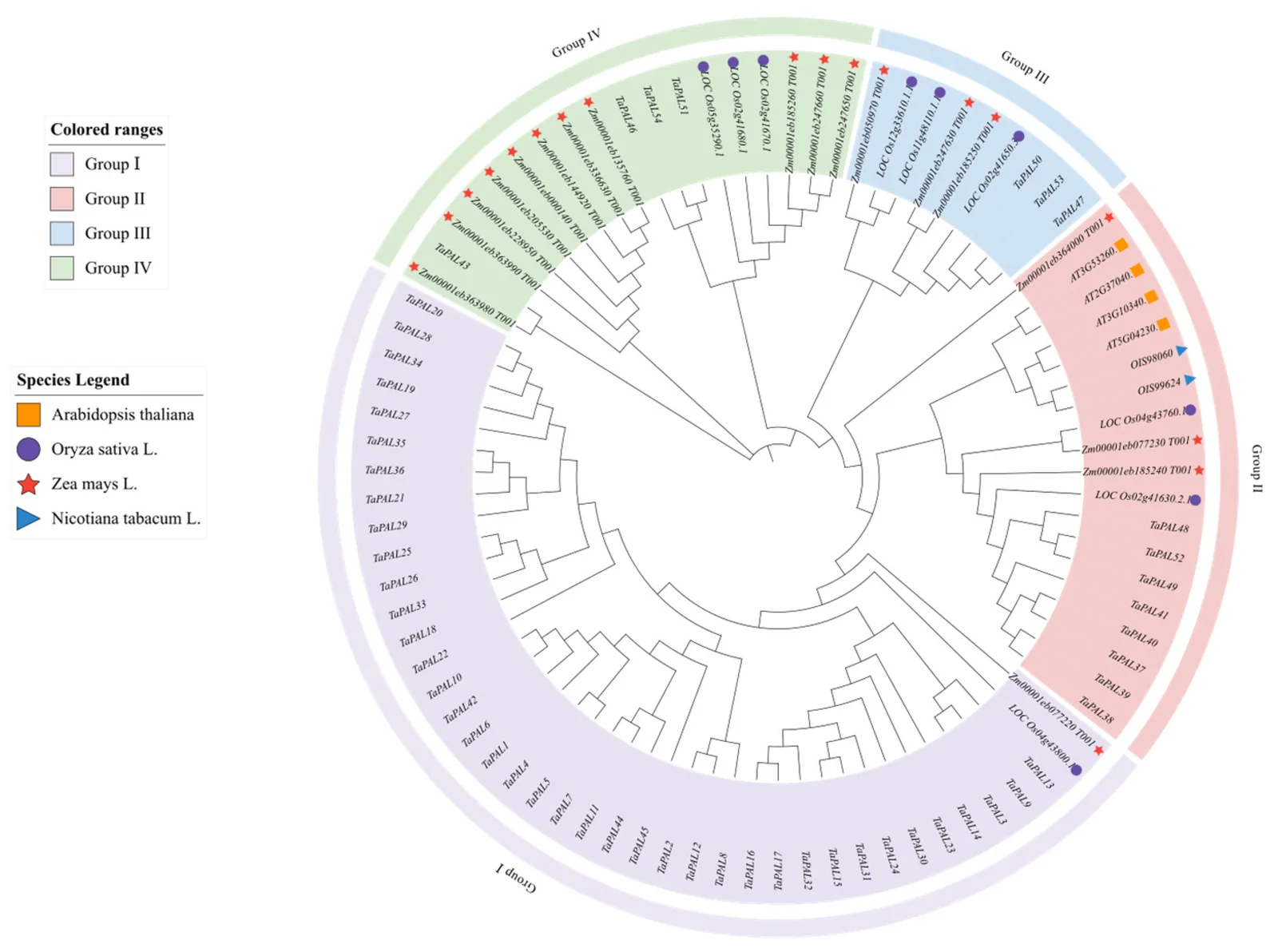
Phylogenetic tree of 87 PAL proteins in five species (wheat, maize, rice, tobacco, and *Arabidopsis*).

**Figure 3:**
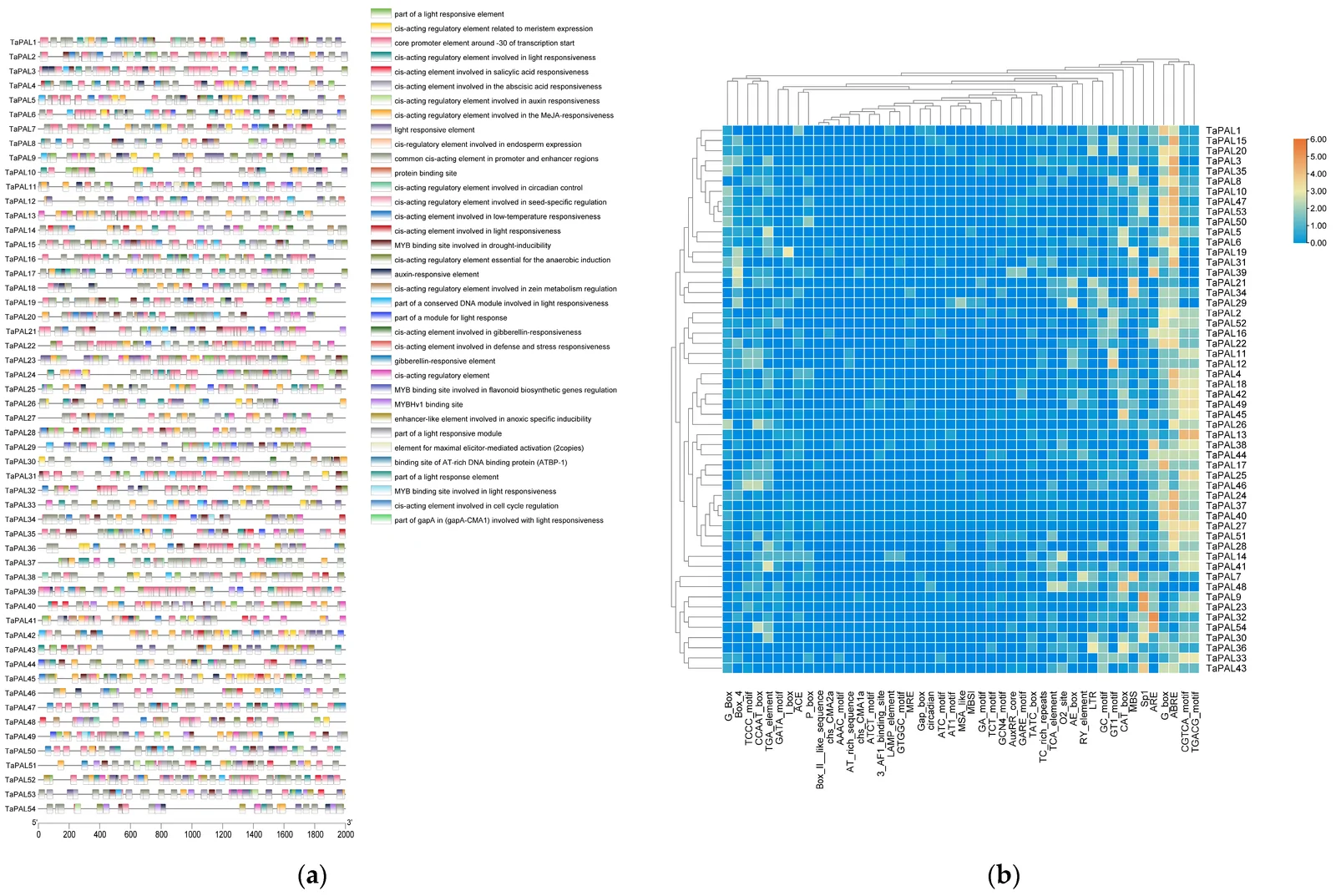
Cis-regulatory element composition of the *TaPAL* gene family. (**a**) Distribution of cis-acting regulatory motifs in the 2000 bp upstream promoter regions of *TaPAL* genes. (**b**) Heatmap showing the abundance of cis-regulatory elements in *TaPAL* gene promoters.

**Figure 4:**
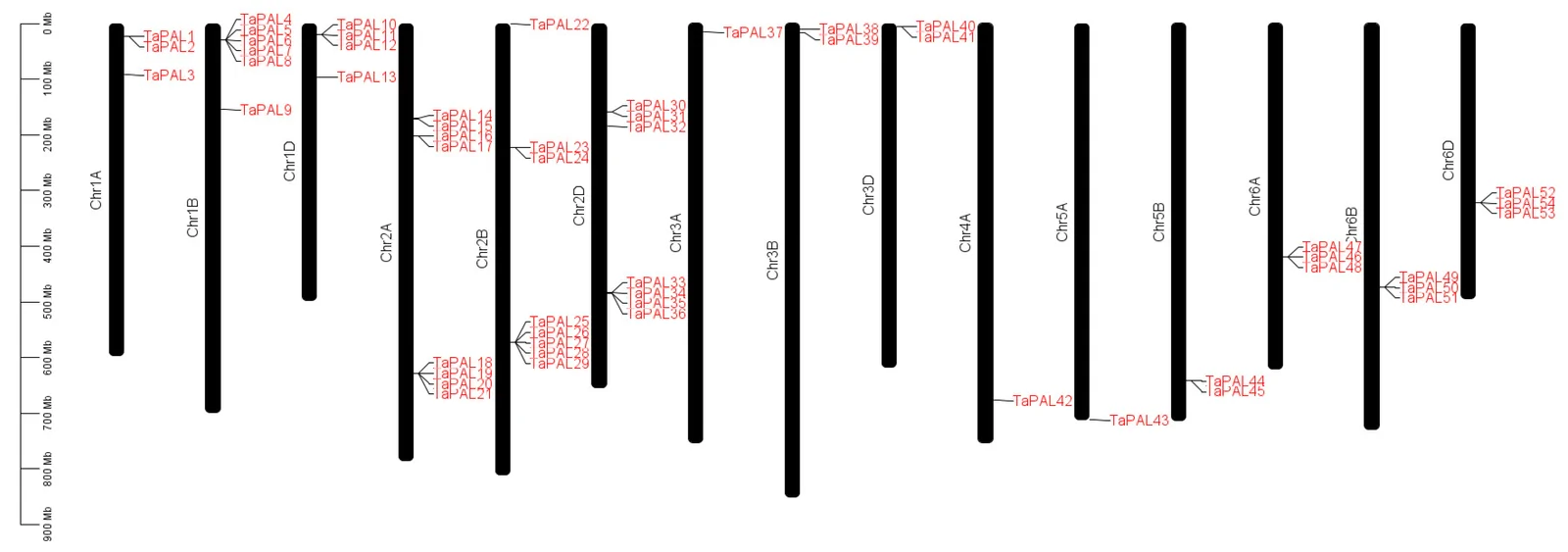
Chromosome distributions of *TaPAL* genes in wheat.

**Figure 5:**
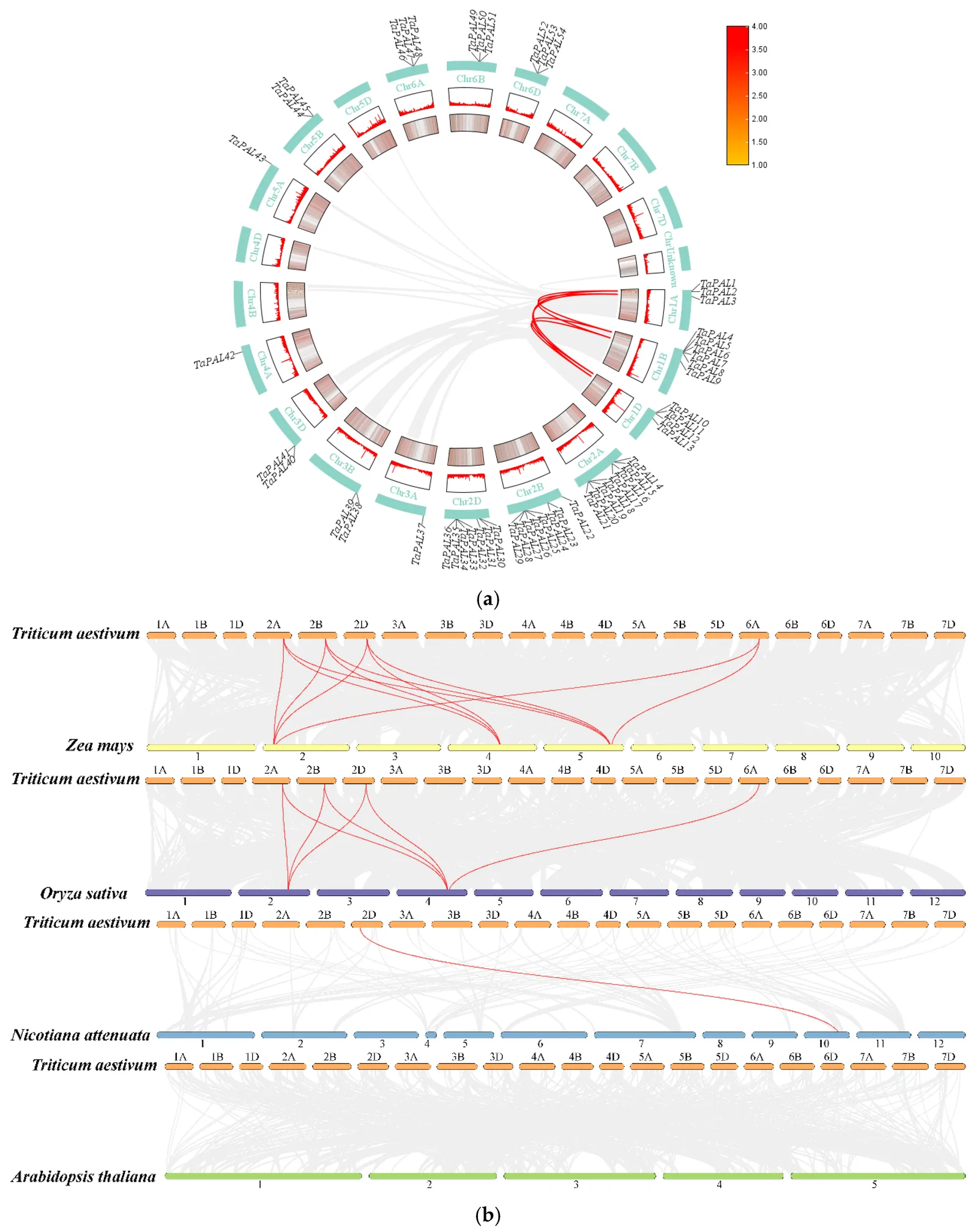
Collinearity analysis of the *TaPAL* gene family. (**a**) Intraspecific collinearity analysis of the *TaPAL* gene family; (**b**) interspecific collinearity analysis of the PAL family.

**Figure 6:**
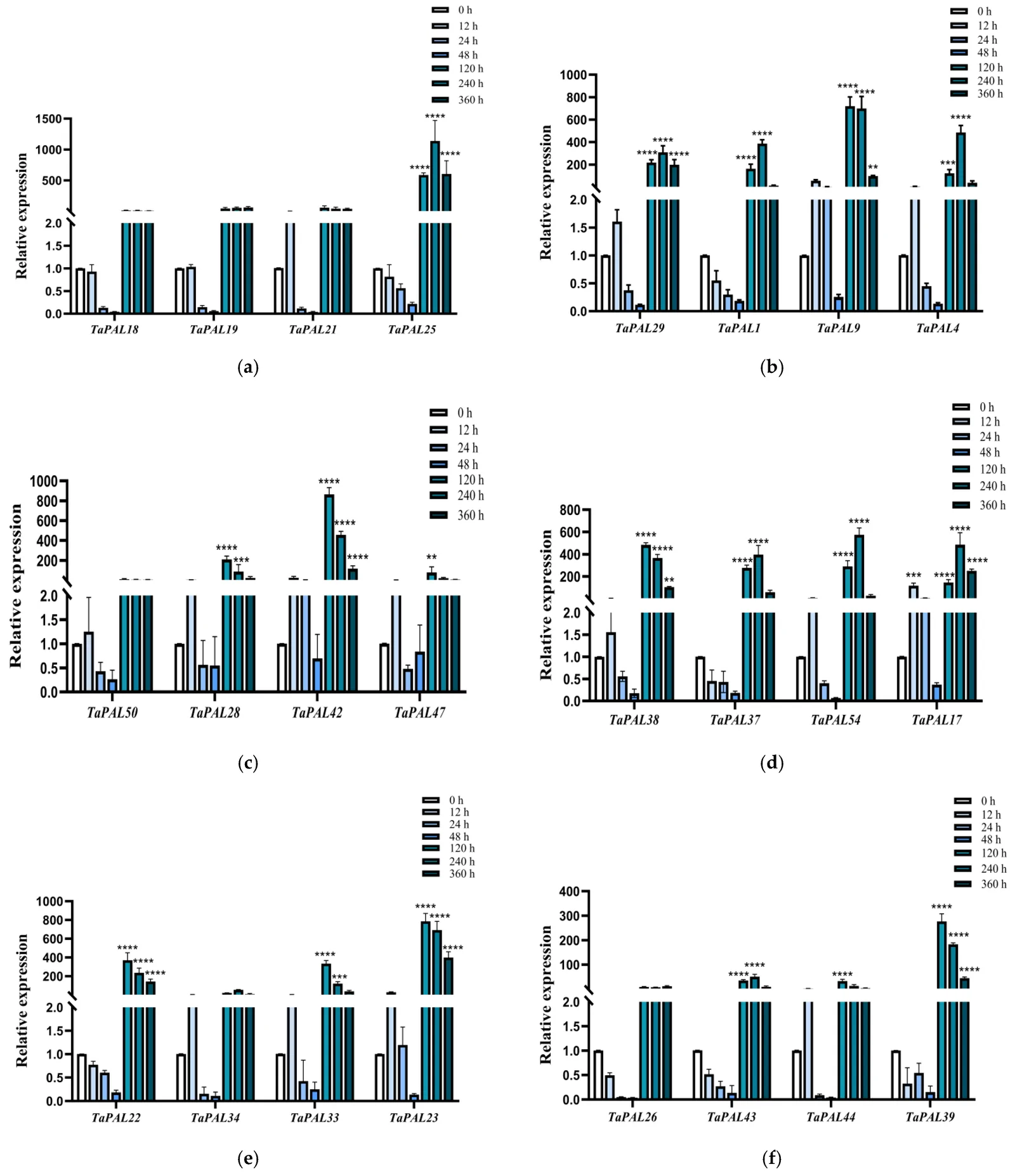
The temporal expression patterns of 24 *TaPAL* genes in wheat following *Pst* infection were analyzed by qRT-PCR. (**a**) The expression analysis of *TaPAL18*, *TaPAL19*, *TaPAL21*, and *TaPAL25* in response to *Pst* infection. (**b**) The expression analysis of *TaPAL29*, *TaPAL1*, *TaPAL9*, and *TaPAL4* in response to *Pst* infection. (**c**) The expression analysis of *TaPAL50*, *TaPAL28*, *TaPAL42*, and *TaPAL47* in response to *Pst* infection. (**d**) The expression analysis of *TaPAL38*, *TaPAL37*, *TaPAL54*, and *TaPAL17* in response to *Pst* infection. (**e**) The expression analysis of *TaPAL22*, *TaPAL34*, *TaPAL33*, and *TaPAL23* in response to *Pst* infection. (**f**) The expression analysis of *TaPAL26*, *TaPAL43*, *TaPAL44*, and *TaPAL39* in response to *Pst* infection. Data are means ± SD (*n* = 3 biological replicates, each with 3 technical replicates). Statistical significance was determined by two-way ANOVA (gene × time) followed by Dunnett’s multiple comparisons test (**: *p* < 0.01; ***: *p* < 0.001; ****: *p* < 0.0001).

**Figure 7:**
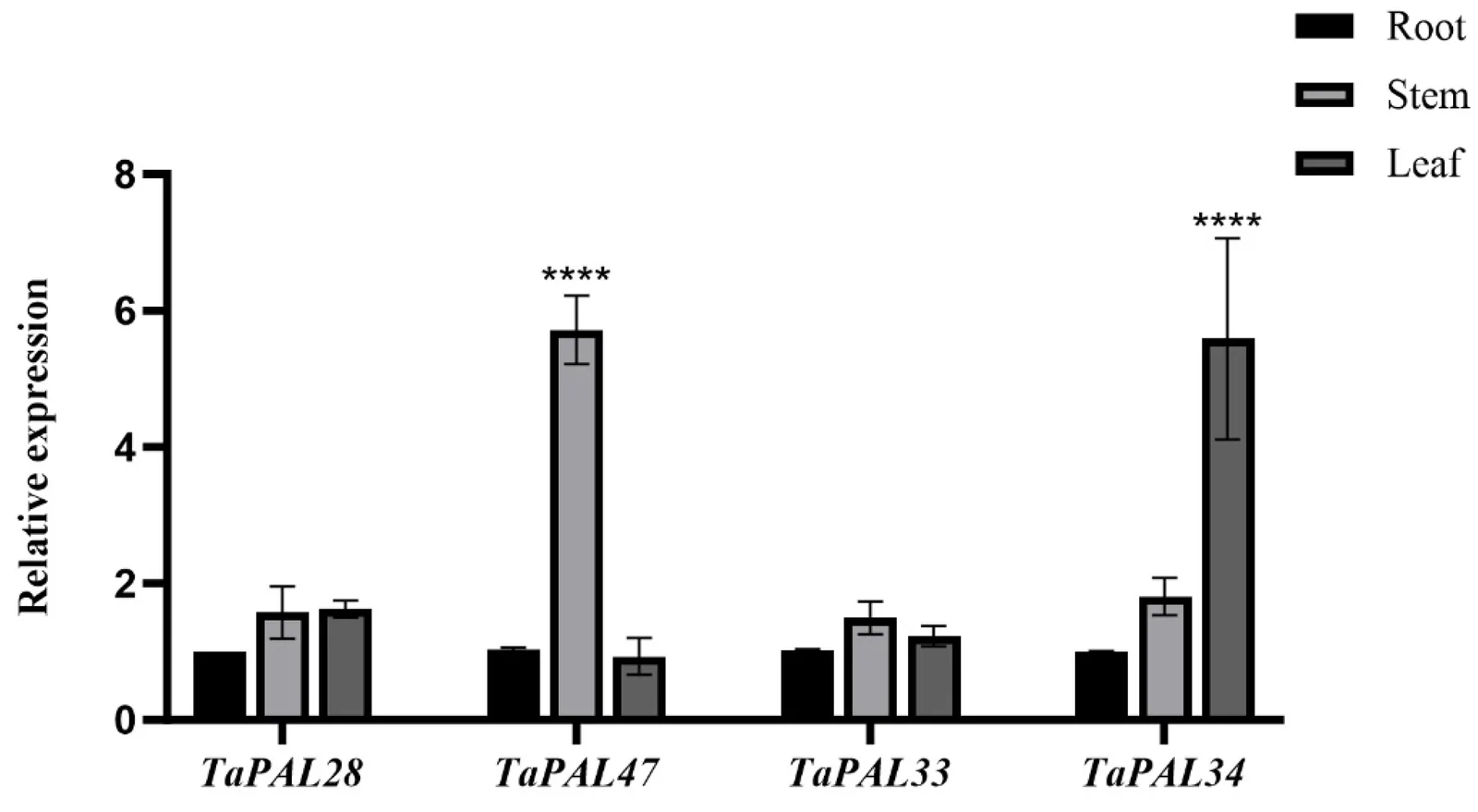
qRT-PCR detection of 4 *TaPAL* genes in different tissues. Data are means ± SD (*n* = 3 biological replicates, each with 3 technical replicates). Two-way ANOVA (gene × tissue) followed by Tukey’s multiple comparisons test was used to compare expression levels among tissues. Asterisks indicate significant differences between the indicated tissues (****: *p*-value < 0.0001).

**Figure 8:**
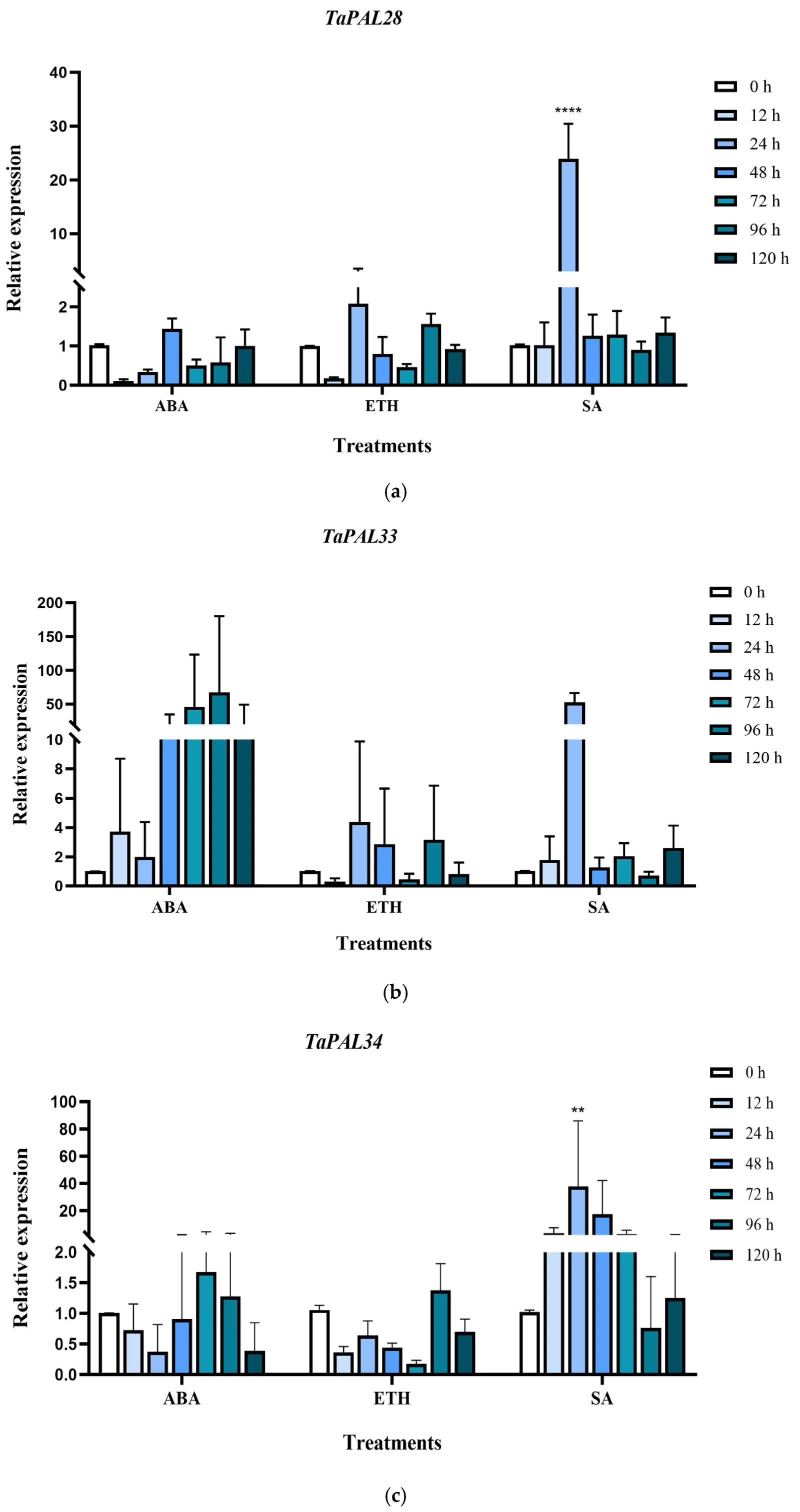

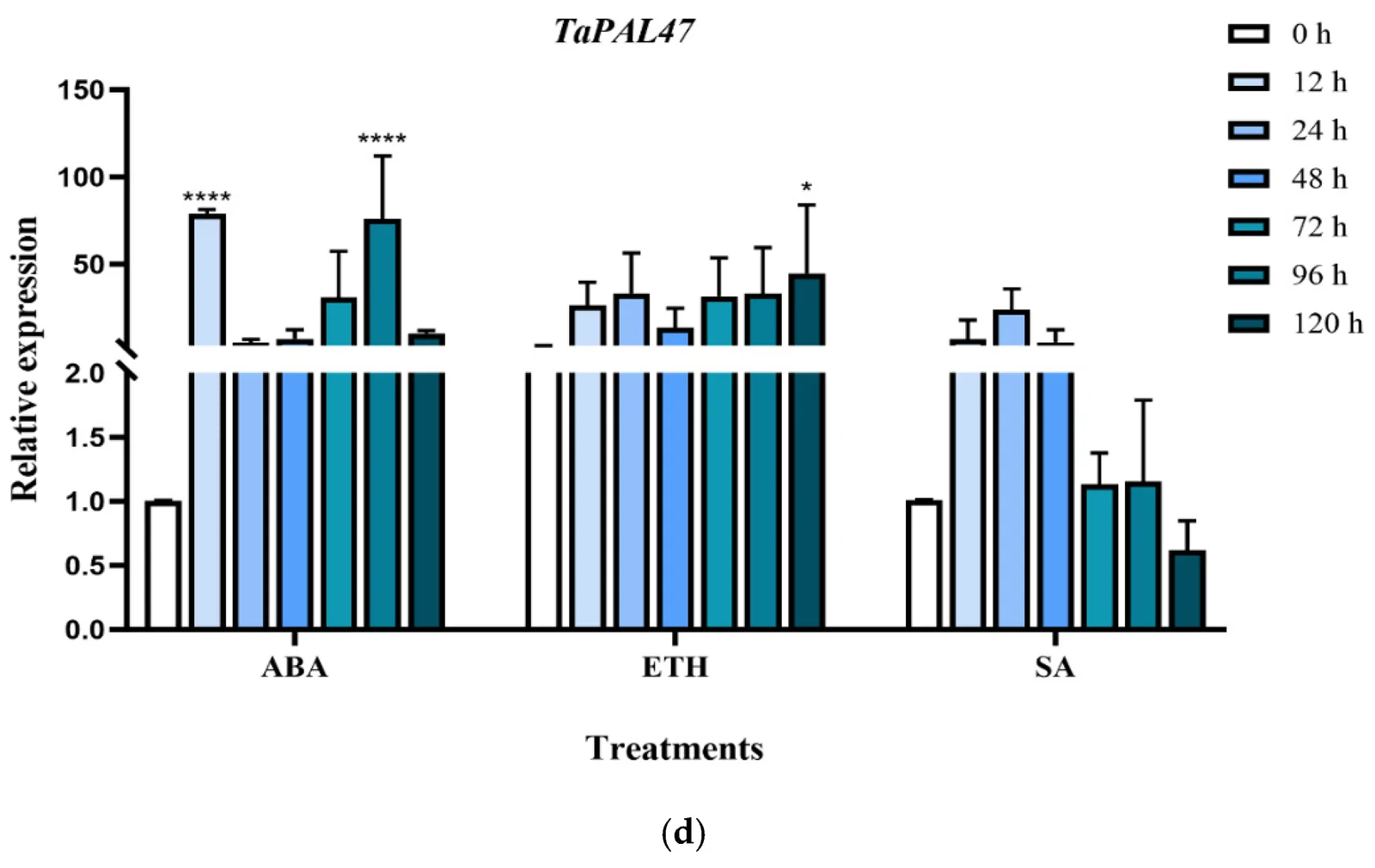
qRT-PCR detection of 4 *TaPAL* genes in response to hormone treatments. (**a**) The expression analysis of *TaPAL28* induced by hormone treatment; (**b**) the expression analysis of *TaPAL33* induced by hormone treatment; (**c**) the expression analysis of *TaPAL34* induced by hormone treatment; (**d**) the expression analysis of *TaPAL47* induced by hormone treatment. Data are means ± SD *(n* = 3 biological replicates, each with 3 technical replicates). Statistical significance was determined by two-way ANOVA (hormone × time) followed by Dunnett’s multiple comparisons test (*: *p* < 0.05; **: *p* < 0.01; ****: *p* < 0.0001).

**Figure 9:**
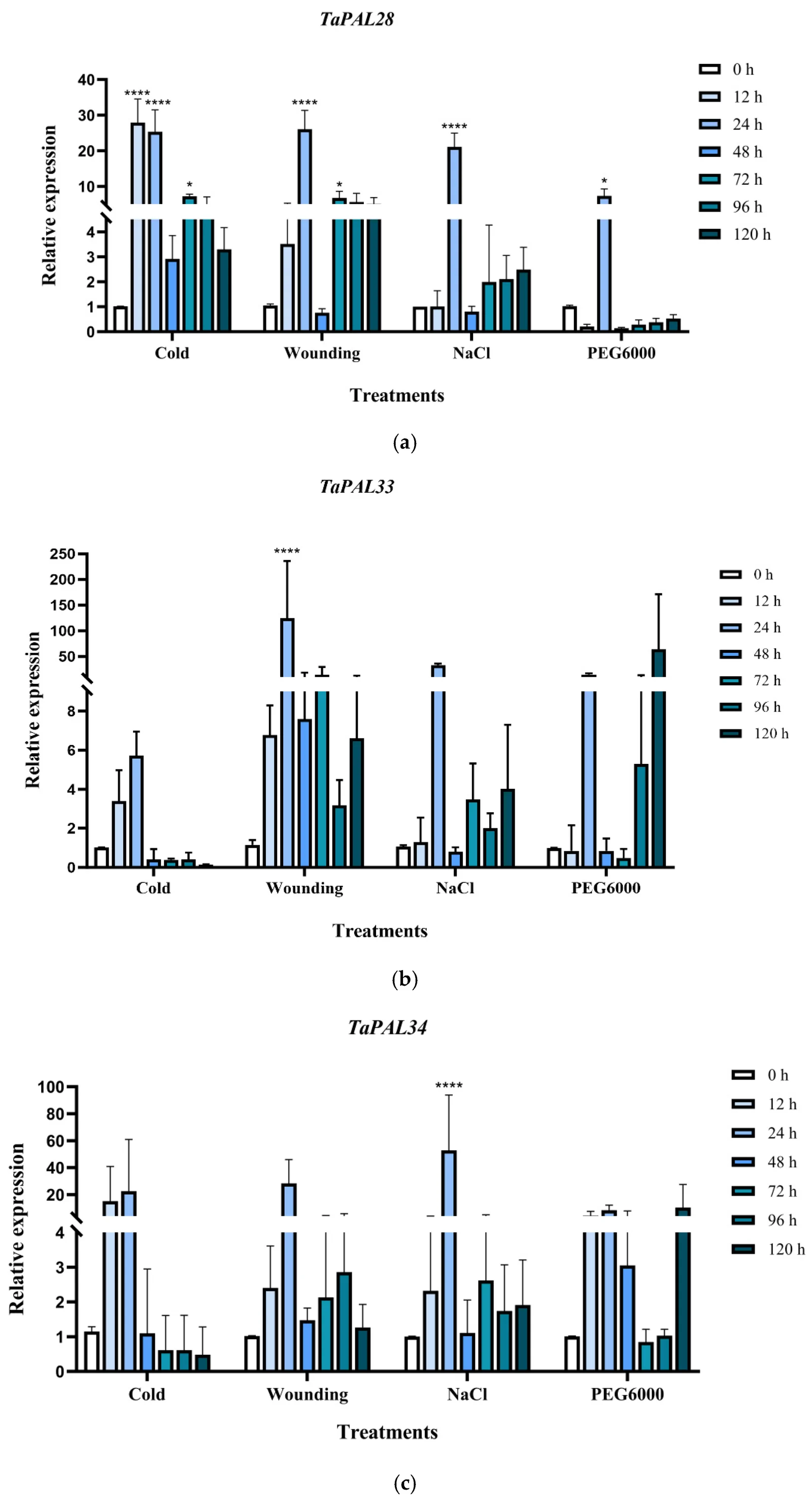

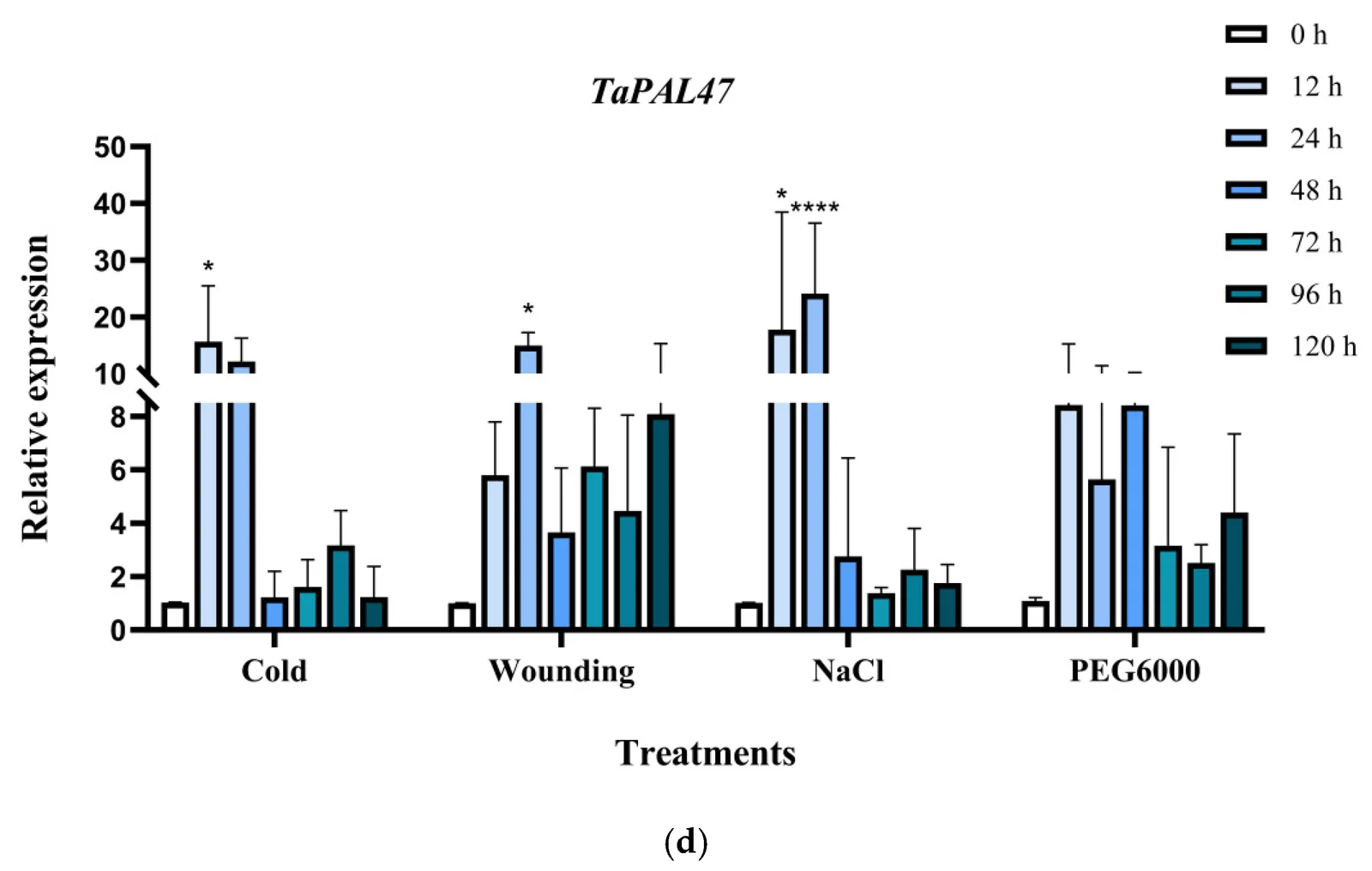
qRT-PCR detection of 4 *TaPAL* genes in response to abiotic stress. (**a**) The expression analysis of *TaPAL28* after treatment with abiotic stresses; (**b**) the expression analysis of *TaPAL33* after treatment with abiotic stresses; (**c**) the expression analysis of *TaPAL34* after treatment with abiotic stresses; (**d**) the expression analysis of *TaPAL47* after treatment with abiotic stresses. Data are means ± SD (*n* = 3 biological replicates, each with 3 technical replicates). Statistical significance was determined by two-way ANOVA (treatment × time) followed by Dunnett’s multiple comparisons test (*: *p* < 0.05; ****: *p* < 0.0001).

## Data Availability

The original contributions presented in this study are included in the article and its Supplementary Materials. All datasets are available in the Supplementary Materials.

## Supplementary Materials

The following supporting information can be downloaded at: https://www.mdpi.com/article/10.3390/biology15191745/s1, Table S1: Genes used in the phylogenetic analysis; Table S2: Renaming of the 54 *TaPAL* genes; Table S3: List of physicochemical properties of *TaPAL* gene in wheat; Table S4: Primer sequences used for qRT-PCR analysis.

## Abbreviations

The following abbreviations are used in this manuscript:

*Pst*	*Puccinia striiformis* f. sp. *tritici*
PAL	Phenylalanine ammonia-lyase
HMM	Hidden Markov model
XC 32	Xinchun 32
ETH	Ethephon
SA	Salicylic acid
ABA	Abscisic acid
hpi	Hours post-inoculation
qRT-PCR	Quantitative real-time polymerase chain reaction
